# Supplementary material for: HIVprotI: an integrated web based platform for prediction and design of HIV proteins inhibitors
Source: J Cheminform. 2018 Mar 9;10:12. doi: 10.1186/s13321-018-0266-y (PMC5845081; doi:10.1186/s13321-018-0266-y)
Supplement: Supplementary file 2 — Additional file 2. Source code of HIVProtI web server. [file 13321_2018_266_MOESM2_ESM.zip › HIVprotI_Source-code/hivprotI/weka-3-6-6/doc/weka/associations/FilteredAssociator.html]

FilteredAssociator


---


|  |  |  |
| --- | --- | --- |
| **Overview** \| **Package** \| **Class** \| **Tree** \| **Deprecated** \| **Index** \| **Help** \| **Weka's home** | |  |
| **PREV CLASS**   **NEXT CLASS** | **FRAMES**    **NO FRAMES**     **All Classes** |  |
| SUMMARY: NESTED \| FIELD \| CONSTR \| METHOD | DETAIL: FIELD \| CONSTR \| METHOD |  |


---


## weka.associations Class FilteredAssociator

```
java.lang.Object
  weka.associations.AbstractAssociator
      weka.associations.SingleAssociatorEnhancer
          weka.associations.FilteredAssociator
```

**All Implemented Interfaces:**: java.io.Serializable, java.lang.Cloneable, Associator, CapabilitiesHandler, OptionHandler, RevisionHandler

---

``` public class FilteredAssociator extends SingleAssociatorEnhancer ```

Class for running an arbitrary associator on data that has been passed through an arbitrary filter. Like the associator, the structure of the filter is based exclusively on the training data and test instances will be processed by the filter without changing their structure.


Valid options are:

```
 -F <filter specification>
  Full class name of filter to use, followed
  by filter options.
  eg: "weka.filters.unsupervised.attribute.Remove -V -R 1,2"
  (default: weka.filters.MultiFilter with
  weka.filters.unsupervised.attribute.ReplaceMissingValues)
```

```
 -c <the class index>
  The class index.
  (default: -1, i.e. unset)
```

```
 -W
  Full name of base associator.
  (default: weka.associations.Apriori)
```

```
 Options specific to associator weka.associations.Apriori:
```

```
 -N <required number of rules output>
  The required number of rules. (default = 10)
```

```
 -T <0=confidence | 1=lift | 2=leverage | 3=Conviction>
  The metric type by which to rank rules. (default = confidence)
```

```
 -C <minimum metric score of a rule>
  The minimum confidence of a rule. (default = 0.9)
```

```
 -D <delta for minimum support>
  The delta by which the minimum support is decreased in
  each iteration. (default = 0.05)
```

```
 -U <upper bound for minimum support>
  Upper bound for minimum support. (default = 1.0)
```

```
 -M <lower bound for minimum support>
  The lower bound for the minimum support. (default = 0.1)
```

```
 -S <significance level>
  If used, rules are tested for significance at
  the given level. Slower. (default = no significance testing)
```

```
 -I
  If set the itemsets found are also output. (default = no)
```

```
 -R
  Remove columns that contain all missing values (default = no)
```

```
 -V
  Report progress iteratively. (default = no)
```

```
 -A
  If set class association rules are mined. (default = no)
```

```
 -c <the class index>
  The class index. (default = last)
```

**Version:**
:   $Revision: 5504 $

**Author:**
:   Len Trigg (trigg@cs.waikato.ac.nz), FracPete (fracpete at waikato dot ac dot nz)

**See Also:**: Serialized Form

---

**Constructor Summary**

`FilteredAssociator()`
  
          Default constructor.


| **Method Summary** | |
| --- | --- |
| `void` | `buildAssociations(Instances data)`             Build the associator on the filtered data. |
| `java.lang.String` | `classIndexTipText()`             Returns the tip text for this property |
| `java.lang.String` | `filterTipText()`             Returns the tip text for this property |
| `Capabilities` | `getCapabilities()`             Returns default capabilities of the associator. |
| `int` | `getClassIndex()`             Gets the class index |
| `Filter` | `getFilter()`             Gets the filter used. |
| `java.lang.String[]` | `getOptions()`             Gets the current settings of the Associator. |
| `java.lang.String` | `getRevision()`             Returns the revision string. |
| `java.lang.String` | `globalInfo()`             Returns a string describing this Associator |
| `java.util.Enumeration` | `listOptions()`             Returns an enumeration describing the available options. |
| `static void` | `main(java.lang.String[] args)`             Main method for running this class. |
| `void` | `setClassIndex(int value)`             Sets the class index |
| `void` | `setFilter(Filter value)`             Sets the filter |
| `void` | `setOptions(java.lang.String[] options)`             Parses a given list of options. |
| `java.lang.String` | `toString()`             Output a representation of this associator |

**Methods inherited from class weka.associations.SingleAssociatorEnhancer**

`associatorTipText, getAssociator, setAssociator`

**Methods inherited from class weka.associations.AbstractAssociator**

`forName, makeCopies, makeCopy`

**Methods inherited from class java.lang.Object**

`equals, getClass, hashCode, notify, notifyAll, wait, wait, wait`

**Constructor Detail**

### FilteredAssociator

```
public FilteredAssociator()
```

:   Default constructor.


**Method Detail**

### globalInfo

```
public java.lang.String globalInfo()
```

:   Returns a string describing this Associator

    :   **Returns:**: a description of the Associator suitable for displaying in the explorer/experimenter gui

---


### listOptions

```
public java.util.Enumeration listOptions()
```

:   Returns an enumeration describing the available options.

    :   **Specified by:**: `listOptions` in interface `OptionHandler` **Overrides:**: `listOptions` in class `SingleAssociatorEnhancer`
    :   **Returns:**: an enumeration of all the available options.

---


### setOptions

```
public void setOptions(java.lang.String[] options)
                throws java.lang.Exception
```

:   Parses a given list of options.


    Valid options are:

    ```
     -F <filter specification>
      Full class name of filter to use, followed
      by filter options.
      eg: "weka.filters.unsupervised.attribute.Remove -V -R 1,2"
      (default: weka.filters.MultiFilter with
      weka.filters.unsupervised.attribute.ReplaceMissingValues)
    ```

    ```
     -c <the class index>
      The class index.
      (default: -1, i.e. unset)
    ```

    ```
     -W
      Full name of base associator.
      (default: weka.associations.Apriori)
    ```

    ```
     Options specific to associator weka.associations.Apriori:
    ```

    ```
     -N <required number of rules output>
      The required number of rules. (default = 10)
    ```

    ```
     -T <0=confidence | 1=lift | 2=leverage | 3=Conviction>
      The metric type by which to rank rules. (default = confidence)
    ```

    ```
     -C <minimum metric score of a rule>
      The minimum confidence of a rule. (default = 0.9)
    ```

    ```
     -D <delta for minimum support>
      The delta by which the minimum support is decreased in
      each iteration. (default = 0.05)
    ```

    ```
     -U <upper bound for minimum support>
      Upper bound for minimum support. (default = 1.0)
    ```

    ```
     -M <lower bound for minimum support>
      The lower bound for the minimum support. (default = 0.1)
    ```

    ```
     -S <significance level>
      If used, rules are tested for significance at
      the given level. Slower. (default = no significance testing)
    ```

    ```
     -I
      If set the itemsets found are also output. (default = no)
    ```

    ```
     -R
      Remove columns that contain all missing values (default = no)
    ```

    ```
     -V
      Report progress iteratively. (default = no)
    ```

    ```
     -A
      If set class association rules are mined. (default = no)
    ```

    ```
     -c <the class index>
      The class index. (default = last)
    ```

    :   **Specified by:**: `setOptions` in interface `OptionHandler` **Overrides:**: `setOptions` in class `SingleAssociatorEnhancer`
    :   **Parameters:**: `options` - the list of options as an array of strings **Throws:**: `java.lang.Exception` - if an option is not supported

---


### getOptions

```
public java.lang.String[] getOptions()
```

:   Gets the current settings of the Associator.

    :   **Specified by:**: `getOptions` in interface `OptionHandler` **Overrides:**: `getOptions` in class `SingleAssociatorEnhancer`
    :   **Returns:**: an array of strings suitable for passing to setOptions

---


### filterTipText

```
public java.lang.String filterTipText()
```

:   Returns the tip text for this property

    :   **Returns:**: tip text for this property suitable for displaying in the explorer/experimenter gui

---


### setFilter

```
public void setFilter(Filter value)
```

:   Sets the filter

    :   **Parameters:**: `value` - the filter with all options set.

---


### getFilter

```
public Filter getFilter()
```

:   Gets the filter used.

    :   **Returns:**: the current filter

---


### classIndexTipText

```
public java.lang.String classIndexTipText()
```

:   Returns the tip text for this property

    :   **Returns:**: tip text for this property suitable for displaying in the explorer/experimenter gui

---


### setClassIndex

```
public void setClassIndex(int value)
```

:   Sets the class index

    :   **Parameters:**: `value` - the class index

---


### getClassIndex

```
public int getClassIndex()
```

:   Gets the class index

    :   **Returns:**: the index of the class attribute

---


### getCapabilities

```
public Capabilities getCapabilities()
```

:   Returns default capabilities of the associator.

    :   **Specified by:**: `getCapabilities` in interface `Associator` **Specified by:**: `getCapabilities` in interface `CapabilitiesHandler` **Overrides:**: `getCapabilities` in class `SingleAssociatorEnhancer`
    :   **Returns:**: the capabilities of this associator **See Also:**: `Capabilities`

---


### buildAssociations

```
public void buildAssociations(Instances data)
                       throws java.lang.Exception
```

:   Build the associator on the filtered data.

    :   **Parameters:**: `data` - the training data **Throws:**: `java.lang.Exception` - if the Associator could not be built successfully

---


### toString

```
public java.lang.String toString()
```

:   Output a representation of this associator

    :   **Overrides:**: `toString` in class `java.lang.Object`
    :   **Returns:**: a representation of this associator

---


### getRevision

```
public java.lang.String getRevision()
```

:   Returns the revision string.

    :   **Specified by:**: `getRevision` in interface `RevisionHandler` **Overrides:**: `getRevision` in class `AbstractAssociator`
    :   **Returns:**: the revision

---


### main

```
public static void main(java.lang.String[] args)
```

:   Main method for running this class.

    :   **Parameters:**: `args` - commandline arguments, use "-h" for full list


---


|  |  |  |
| --- | --- | --- |
| **Overview** \| **Package** \| **Class** \| **Tree** \| **Deprecated** \| **Index** \| **Help** \| **Weka's home** | |  |
| **PREV CLASS**   **NEXT CLASS** | **FRAMES**    **NO FRAMES**     **All Classes** |  |
| SUMMARY: NESTED \| FIELD \| CONSTR \| METHOD | DETAIL: FIELD \| CONSTR \| METHOD |  |


---
